# On the Common Origin of All Varioloid Eruptions

**Published:** 1820-08

**Authors:** Rod. Macleod

**Affiliations:** Physician to the Westminster General Dispensary.


THE LONDON

### Medical and Physical Journal.

2 OF VOL. XLIV.] AUGUST, 1820. [NO. 258.

"For many fortunate discoveries in medicine, and for the detection of numerous "errors, the world is indebted to the rapid circulation of Monthly Journals; "and there never existed any work to which the Faculty in EUROPE and "AMERICA were under deeper obligations than to the Medical and Physical "Journal of London, now forming a long, but an invaluable, series." RUSH.

### Original Communications, Select Observations, etc.

FOR THE LONDON MEDICAL AND PHYSICAL JOURNAL.

### *On the Common Origin of all Varioloid Eruptions.* By ROD. MACLEOD, M.D. Physician to the Westminster General Dispensary.

WHAT is meant by small-pox, we all know pretty well: what is meant by chicken-pox, does not seem quite so easily determined; the reason of which probably is, because that name has been given to forms of eruption essentially different from each other. Most medical men have a definite idea of small-pox; and, when they meet with an eruption which is too mild in itself, or too short in its duration, to come up to their idea of that disease, they satisfy themselves that it must necessarily be chicken-pox. But the truth is, that the varioloid disease, both in the vaccinated and unvaccinated, assumes every variety of form, occupying all the intermediate stages between the mild and the malignant, the simple vesicular, and confluent pustular, eruption. I lately attended a family in Hungerford-street, none of whom had been vaccinated: in the eldest the disease assumed the confluent form, and proved fatal; the youngest had a vesico-pustular eruption, consisting of only six pocks, which did not affect the general health for an hour. Two others of the family exhibited intermediate degrees; one approaching the malignant, the other the mild, eruption. Two of these cases, if they had occurred sporadically, would, I am convinced, have been regarded as chicken-pox. Thus, I believe, in common language, variola and varicella are terms denoting the extreme points of two forms of eruption, so similar in kind, and passing so insensibly into each other, that it is impossible to fix the exact limits of either. To show differences between these extreme points when there is in reality no resemblance, is a work of supererogation; which, nevertheless, some authors have taken upon themselves: the difficulty and the failure has been, in endeavouring to determine how much of the pustular character an eruption may assume, being still chicken-pox; and how much of the mild vesicular character, being still small-pox. Under these circumstances, it is not to be wondered at that medical practitioners, having different notions with regard to the degree of severity which constitutes each disease respectively, should differ so widely in their opinions; for it is notorious, that what one man calls variola another very often holds to be varicella.

Heberden, who first attempted to point out and define a certain form of varioloid eruption, as specifically distinct from the others, says of the variolæ pusillæ, "Principio rubræ sunt *postridie* in earum apicibus exigua vesicula est." Mr. Moore, in one place, speaks of the "chicken-pox pimple;" and in another informs us, "that each little tubercle is smaller, flatter, and less painful, than in small-pox." Willan says, the sensation communicated to the finger in varicella, is like that from a round seed flattened by pressure;" and Dr. Monro, that "some of the pimples become larger, and sometimes pustular, after the fourth day." I make these quotations, in order to show that chicken-pox has hitherto been described as an eruption which, in its commencement at least, is papular. Mr. Bryce, on the other hand, says, "The vesicles are often, when first seen, about the size of a spilt-pea, perfectly transparent, and covered only by a cuticle as thin as that separated by a scald or by a blister. They generally have at first an inflamed areola; but this seems also *to be confined to the cuticle, and there seems to be little, if any, hardness in the true skin beneath or around them.*" So likewise Dr. Abercrombie: "When a single specimen of the eruption is examined, it is found to be *from the earliest period a watery vesicle.*" "If one of the vesicles of this eruption be punctured on the second or third day, so as carefully to discharge all the fluid, the pellicle falls down, and, the finger being carried over it, it is found to be correctly on a level with the surrounding integuments." That an eruption, which only becomes vesicular on the second day, according to Heberden; in which there are little tubercles, according to Moore; and induration resembling a seed flattened by pressure, according to Willan; is not the same as one which is vesicular from the earliest period, seems, *prima facie*, sufficiently obvious; and I should not have thought it necessary to point out the difference, had not the reviewer of Dr. Thomson's work, already alluded to, informed us that they agreed "exceedingly well." I am inclined however to think, that this gentleman, whose view of the subject is certainly ingenious, has nevertheless committed an oversight, which implies a contradiction of the opinion here expressed by him. Dr. Thomson relates, at page 181, a case, in which he asserts the eruption to have been, even from its commencement, purely vesicular, and therefore chicken-pox. The reviewer argues, that it was not the chicken-pox of Mr. Bryce, &c. and says in a note, "The only part of the history of the above case, as given in Dr. Thomson's work, which we should suspect not to be perfectly accurate, is the statement of the eruption having been *purely vesicular* from the first. From our observation of the appearance of the eruption in this case, we should be inclined to suspect that it must have been papular for some little time (perhaps twenty-four hours) before it became vesicular." Now, whether Dr. Thomson was right or not in his opinion of the case alluded to, is foreign to the present question: what I wish to illustrate is, that the reviewer himself seems, perhaps unconsciously, to have made a distinction between the chicken-pox of Heberden and that of Bryce, &c.; for it is a palpable inconsistency, to assert that a description which expressly states varicella to be in the first instance papular, agrees with one which makes that disease so purely vesicular, that even the conjecture of this case having been papular "for some little time, perhaps twenty-four hours," formed one of the reasons which prevented him from regarding it as true chicken-pox. Another difference is, that the chicken-pox of Heberden and Willan was capable of being communicated by inoculation; whereas the Edinburgh chicken-pox is incapable, so far as experiments have yet been made, of being communicated in this way.

In the days of Heberden, small-pox after small-pox was supposed to be so rare, that its very existence was doubted; and, consequently, an eruption subsequent to variola, was regarded as necessarily something different; whereas more recent observation has proved the occurrence to be by no means very uncommon. Does not this, in conjunction with the fact that the papulo-vesicular form of eruption is capable of communicating true small-pox to the unprotected, tend to favour the idea that Heberden, and other writers upon this subject, described cases of small-pox mitigated by the previous existence of that disease, or by other circumstances, such as sometimes render the eruption communicated by confluent small-pox perfectly mild and varicelloid? This idea is very distinctly set forth, in an essay upon Spurious Small-pox, published several years since at Gottingen, as will appear from the following quotation. Referring to another part of the work, it is said, "Attamen cum istas varicellas in illis qui eam ob causam quia variolis jam laborarunt dispositione carent potissimum prodire illuc adtulerim, non possum quin Hufelandi observationibus nisus ejusmodi varicellas ex dispositione deficiente in illis quoque qui variolis nondum laborarunt et tamen dispositione carent oriri posse hic indicem."*

Of late years, when a suspicious eruption occurred in a person who had been vaccinated, it was regarded as a legitimate inference that such eruption, at all events, could not be variola. Now when we consider the very summary process of ratiocination adopted on such occasions, does it not appear likely that practical writers, in describing varicella, had in view, at least in some instances, cases of small-pox reduced to the papulo-vesicular form by previous vaccination; and so laboured in vain to establish a difference where no difference existed. The admission of this position would, I think, be greatly in favour of cow-pox; inasmuch as it would show, that the proportion of cases in which small-pox occurs after vaccination, is not really increasing so much as has recently been feared, but only that more accurate observation and experiment had proved an eruption, which we have regarded as specifically different, to be in fact mitigated small-pox. Certain it is, that papulo-vesicular eruptions, so mild that they would very lately have passed unquestioned as cases of chicken-pox, do, by inoculation, communicate small-pox to the unprotected. Such for instance is the case of Henry Oldfield, related in my former paper, where vesicles were formed on the second day, and from whom two unvaccinated children had small-pox communicated by inoculation; as the Editor of the Medical and Physical Journal, who frequently saw them, can testify. My reasons for performing this experiment were, that their mother would not consent to have them vaccinated; that, as they were unavoidably exposed to the contagion from residing in the same house, it was better they should have the disease communicated by inoculation than take it naturally; and because, if the eruption was really only varicella, little danger was likely to be incurred.

Nothing can more clearly show the insufficiency of the attempts hitherto made to establish a satisfactory diagnosis between small-pox and chicken-pox, than the fact that Mr. Moore, whose opinions on such subjects, from his official situation furnishing him with so many opportunities of investigation, may be regarded as authority, acknowledges, at page 109 of his work on Vaccination, that cases are met with in which it is altogether impossible to distinguish between them, by any appearances assumed by the eruption, "from its commencement until its termination." In this dilemma, he suggests that assistance may be derived from the presence or absence of cough, which, he says, has nothing to do with small-pox, and from the degree of constitutional derangement. With regard to the first

* Gustavi Lavr. Julian Muhrbeck, *Dissertatio Medica de Variolis Spuriis.*

of these: if two mild cases of a doubtful eruption occurred, alike in all other respects except that one patient had coughed and the other had not, would any practitioner infer from this, that one had got variola, and the other varicella? Besides, cough occurs very frequently in true small-pox, and, in fatal cases, is satisfactorily accounted for by the inflammation of the mucous membrane of the bronchiæ, so generally found on examination after death. As to the constitutional derangement, it is necessarily so indefinite as to afford but little assistance; for no one has yet ventured to fix the exact degree which constitutes an eruption small-pox, and *vice versa.*

Another method, however, of distinguishing between these eruptions has recently been suggested: I mean the anatomical structure of the pock. This, though it has but lately attracted much notice, was described by some of the older writers; but by none, I believe, so fully as Cottunius, in his work "De Sedibus Variolarum," in which the subject is illustrated with a plate. More minute, and probably more accurate, observations have been made by some pathologists of the present day.* It is asserted, that, in true variola, the pock is in its early stage cellular; that these cells are gradually broken down, or otherwise disappear, so as to convert the whole into a simple bag, in the bottom of which a slough is to be found. With regard to the cellular texture, it is not, I imagine, very easily demonstrated; and I cannot help doubting whether it invariably exists. I have tried the experiment of making a minute puncture in vesico-pustular eruptions arising from small-pox, and have, in such instances, generally seen the fluid almost instantly evacuated; a circumstance which, I apprehend, could not have happened, if it had been obliged to pass from one cell into another on its journey to the opening: a phenomenon strikingly illustrating the cellular nature of the cow-pox vesicle. In severe small-pox a slough is certainly to be found, and its occurrence necessarily gives rise to loss of substance, and consequent pitting; but, as slight cases of small-pox do not give rise to pitting, so it would appear, that the formation of a slough, though a frequent, is not a necessary, attendant upon a truly variolous disease. But, as it is only in distinguishing between mild cases and chicken-pox that any doubt can arise, so it really seems difficult to conceive how this piece of pathology, however interesting in itself, can ever be of use in a diagnostic point of view. There is another objection to a diagnosis founded on anatomical structure, viz. the inconvenience of its application: indeed, it would not be far removed from the ridiculous, to see a physician, on being

* Since these remarks were written, the valuable work of Mr. Cross has been published.

asked the nature of an eruption, pull out his microscope and lancet, and cut open the pock, as he would examine a plant, to determine its class and order.

But, granting that in some forms of eruption we always find a slough, and in others we never do, it remains to be shown how this proves a specific difference between them. The external appearance alone sufficiently indicates very different degrees of inflammation in different eruptions: when the inflammatory action is confined to the rete mucosum, or surface of the true skin, then it quickly terminates in the effusion of serum; if it be more severe, then the surrounding textures become involved to a certain extent, and pus is formed, with occasional ulceration; lastly, if the inflammation runs high, it ends in the death of the part. These effects bearing an exact relation to the degree of inflammation, is it not reasonable to suppose they depend wholly upon it? and, consequently, that the presence or absence of slough only indicates differences in the degree of inflammation, not in its nature? A blister excites inflammation generally terminating in serous effusion; sometimes, however, it runs on to ulceration, or even sloughing: yet we never hesitate in attributing all these effects to the same exciting cause. So likewise mercury, taken internally, sometimes causes erythema, ending in vesication, and sometimes a pustular eruption. May it not be the same with the variolous poison, which, when applied to the human system, has its effects modified by various constitutional, and other yet more inexplicable causes?

I have already mentioned, that it seemed admitted on all hands, in Edinburgh, that the form of eruption which was in its commencement papular, both arose from, and reciprocally gave rise to, genuine small-pox, and consequently was itself to be regarded as truly variolous. This was probably the circumstance which suggested to Mr. Bryce, Dr. Alison, and Dr. Abercrombie, the propriety of restricting the term varicella within narrower limits, viz. to an eruption purely vesicular; a form in which, they maintain, it is not capable of being propagated by inoculation, and in which it runs a course independent on, and unconnected with, small-pox. With regard to the first of these characteristics, it is obviously as yet matter rather of presumption than demonstration; and, secondly, even if proved, it would by no means follow as a necessary consequence, that it arose from the contagion of a poison specifically different from small-pox, as it is very easy to conceive that the fluid never attains the degree of maturity necessary to communicate the disease. Thus, we have the authority of Dr. Willan for asserting this of another morbid poison, viz. cow-pox. He informs us, at page 32 of his work on Vaccine Inoculation, that, if lymph be taken from a perfect vesicle too early, that is, on the sixth or seventh day, "it does not always produce the genuine cellular vesicle, but is sometimes wholly inefficient." So likewise Mudge informs us, that a woman at Plymouth was inoculated for small-pox, and that, five days after, thirty men were inoculated from her arm before the matter was matured; while, in ten, the operation was not performed till maturation had taken place: these ten took the small-pox; but, in those first inoculated, a local affection only was produced, though, on repeating the operation again, they all took the disease. Are we not justified in inferring from these authorities, that some certain degree of maturity is required, to enable the poisons of cow-pox and small-pox to communicate similar diseases by inoculation, and that the mildness of the inflammatory action, and consequent shortness of the vesicular eruption, prevents this maturation from being effected?

With regard to the second part of the question, in which this purely-vesicular disease is asserted to run its course unconnected with small-pox, it would be foreign to the purpose of these remarks to enter into the discussion which has occurred in Edinburgh. The great prevalence of the disease in the northern metropolis, though it afforded excellent opportunities of establishing its characters, yet had the disadvantage of rendering it more difficult to trace the sources of contagion: for, as it seems to have prevailed in every form, in all the courts and alleys, and almost in every house, it is impossible to feel absolutely sure that the vesicular did not generally arise from the vesicular, and the pustular from the pustular, eruption. In London, the disease not having been so universal, it is more easy to trace in a satisfactory manner the probable source of contagion. The reviewer of Dr. Thomson's work, who has evidently watched the disease in Edinburgh with an observing eye, allows that, if we could find an eruption distinguished in its early stage by Mr. Bryce's marks of chicken-pox, manifestly giving rise, even in a single instance, to unequivocal small-pox in an unprotected child, or to an eruption distinguished in its early stage by Dr. Abercrombie's marks of horn-pocks, in a vaccinated child, we should consider the case as nearly decisive in Dr. Thomson's favour. Now I am inclined to suppose, *mutatis mutandis*, that it must be equally in favour of the identity of the two diseases, if we can show unequivocal small-pox giving rise, in a single instance, to the Edinburgh chicken-pox; and I submit how far the following cases tend to illustrate this idea.

Caroline Wallace, aged five years, residing at No. 4, South-street, Grosvenor-square, has a perfect vaccine cicatrix on the arm. Shortly after exposure to the contagion of small-pox in its natural form, she took a varioloid disease, in the beginning of March, the description of which corresponded with that of the papular form of small-pox, as it frequently occurs after vaccination. This child, while labouring under the eruption above described, was in the habit of being in the room and playing with Edwin Hollis, aged fifteen months, and likewise vaccinated, who became affected, March 9th, with a vesicular eruption, without any previous appearance of papulæ: next day the vesicles were becoming opaque, and on the third began to shrink. Fresh ones however continued to come out, which, like the former, consisted of a mere separation of the cuticle from the cutis. On being punctured, a drop of clear fluid was immediately evacuated, and the skin fell so flat, that neither Dr. Gregory nor I could detect any induration, on running our fingers over the part. This child was the patient of my friend Dr. George Gregory.

A child, aged eight weeks, residing in Peter-street, and who had never been vaccinated, became affected with small-pox, which was at its height May 16th. In the adjoining house is a shop, to which two little brothers of this infant were in the habit of going, and a school. On the 18th, a child, residing in the floor above the school-room, who has a perfect vaccine cicatrix upon the arm, became affected with a varioloid eruption, which, at the twelfth day, presented the appearance of large flat crusts surrounded with considerable induration.

Diana Corbet, aged six years, not vaccinated, attending the school, next caught the disease in a mild form, and brought it to No. 8, Silver-street, Golden-square, where she resided. Her sister Sarah, aged four months, not vaccinated, took a more severe eruption, which is said not to have come to its height in less than a week.

In the adjoining room to the Corbets were two children, who constantly associated with them. John Hutchins, aged eight months, inoculated at Broad-street, but without taking the cow-pox, became affected with an eruption, May 20th, which is described as having consisted at first of pimples, and subsequently become pustular; the pocks, to use the mother's expression, being "dented down in the middle." This eruption retained, on the tenth day, much of the tubercular appearance so frequently succeeding to mild small-pox. Maria Hutchins, aged three years, vaccinated, when seven or eight months old, at the Institution in Broad-street, and having four perfect cicatrices on each arm. May 30th. Became affected, a few days ago, with an eruption of vesicles like little blisters, some of which have shrunk, and none of which present anything of the papular character. Fresh vesicles continue to come out, particularly about the arms and thighs: they were about the size of a splitpea, flat, roundish, but not exactly circular; and they have scarcely any perceptible inflammation, and no perceptible induration round them. On being punctured, clear lymph is evacuated, and the skin falls flat down, looking like little white stains, or small pieces of thin writing-paper laid upon the part. 31st. Some of the vesicles giving place to little flat shining crusts: no fresh ones have appeared since yesterday. The fluid evacuated on puncturing the vesicles is now like milk, and my friend Dr. Richard Harrison, on running his finger gently over the part, with his eyes shut, cannot detect the site of the pock. In a few days after the date of this report, the eruption died away in the form of scabs, which, on dropping off, left no marks.

In the beginning of January, the small-pox, in its unmodified form, prevailed at No. 26, New Compton-street. At this time, three children named Lucas, residing in the adjoining house, No. 25, all of whom had been vaccinated, became affected with a varioloid disease. In the eldest, who took it first, the eruption is said to have begun in the form of pimples; by the time I saw him it had passed into the vesico-pustular state, and ran its course within a week. In the youngest, who took it next, it had but little of the papular character; but successive crops continued to come out, so that the duration of the eruption was protracted to about a fortnight. In the third, Benjamin, aged two years, the eruption is described in my notes as having consisted in a few vesicles, viz. one on the nose, three over the left eye-brow, and five or six on the chest: these seemed to be formed "by a separation of the cuticle from the cutis, filled with clear water, and without any surrounding hardness or inflammation; some of them are broader at the top than at the base, and they have nothing like central depression." Successive vesicles appeared for several days, but none, individually, lasted for more than three days without shrinking and becoming covered with a crust. After these crusts, or scabs, were formed, some induration took place; and it would now, I believe, have been very difficult to have distinguished this from a very thin crop of the tubercular form of the varioloid eruption called horn-pock. I remember pointing out this case to the pupils at the Dispensary, as agreeing in every particular with varicella, as described by Mr. Bryce, &c. from the striking resemblance it bore to little blisters from drops of boiling-water.

I heard nothing more of these children till May, when I was again requested to see the eldest, Thomas, who laboured under a second attack of varioloid disease. Small-pox now prevailed in its unmodified form in the next house but one, No. 23, and in one instance proved fatal. In this house was a child pitted all over from a previous attack of small-pox, with whom Thomas

Lucas was in the habit of associating and playing about the door of No. 23, when the child who died lay ill of the small-pox. These two children both became affected on the same day with an eruption, which in Lucas proved papular, vesicular, and pustular, as described at length, Case No. I. The case of the other was as follows:

Martha Rashwood, aged six years, pitted with small-pox, which she had a year and a half ago in a severe form, never having been vaccinated. May 17th. Was observed yesterday to have some little red points and vesicles in different parts of the body; was sick and heavy this morning, and a pretty copious crop of small vesicles has made its appearance. They have but a very slight blush of inflammation round them, and, on being punctured, fall flat, so that nothing like papular induration can be felt on running the finger over them. 18th. Some of the vesicles have burst and shrunk; no hardness to be perceived; fluid in the vesicles slightly opaque. 19th. Vesicles have almost all given place to little flat crusts. Dr. Ashburner, on dissecting a pock this day, with much care, cannot detect the slough which he regards as characteristic of small-pox.

Benjamin Lucas, aged one year and three months, sleeping with his brother Thomas, has little red points come out on different parts of his body, May 21, which in two days were nearly as large as a split-pea, with the slightest imaginable elevation, rather to be detected by running the finger over them, than by their appearance. These in three or four days disapp r d, without any vesication. Is the occurrence of these spots to be regarded as merely accidental, or as an attempt at varioloid action, which was overcome by the guarded state of the constitution?

That varicella frequently occurs sporadically, is known to every one; but I never have seen an eruption occurring in that way answer the description of chicken-pox, as given by Mr. Bryce, more entirely than the cases above related, in which it was associated with small-pox. Such association does not demonstrate, indeed, the identity of their origin; but the frequency of its occurrence amounts to presumptive evidence in favour of that opinion. In the case for instance of Ann Rashwood, it would be pushing the opposite doctrine to a length not warranted by any probability, to argue that the eruption might have arisen from some other contagion, and not from the small-pox in the house. But, if one single case be admitted, of true varicella arising from true variola, it proves that they do not necessarily arise from separate specific poisons just as completely as if we could bring an hundred such instances.

It is a mistake, to suppose that the doctrine of the common origin of all the various forms of varioloid eruption, is new; for it was maintained by some continental writers before the discovery of cow-pock, and in opposition to the opinion of Heberden. "Varicellæ exanthemata sunt acuta, variolis veris, respectu formæ et decursus plus minusve similia, harumque miasmate vel corrupto, vel non rite evoluto, vel corpore ad recipiendum miasma indisposito, exorta, ita ut à variolis non præservent, decursumque breviorem teneant." And again, "Variolas itaque perniciosissimas et varicellas levissimas ex eodem primitus gigni fomite, &c."* In this quotation it is stated, that chicken-pox does not guard the system against small-pox; but, as it has recently been proved that small-pox does not secure the constitution against its own recurrence, it is obvious that the arguments, which some are disposed to draw from this circumstance against the identity of the diseases, are wholly inadmissible.

Are the following conclusions warranted by the foregoing remarks:

That there is no form of varioloid or varicelloid eruption hitherto described, which does not, under particular circumstances, arise from the contagion of small-pox?

That small-pox after vaccination has never been so rare as we supposed, because the circumstance of previous vaccination was till lately regarded as sufficient proof that subsequent eruptions were not variolous?

And that practical writers, particularly those who have described varicella as an eruption in its commencement papular, have only been describing small-pox, modified by vaccination or other circumstances?

*Great Marlborough-street; May* 1820.

---

FOR THE LONDON MEDICAL AND PHYSICAL JOURNAL.

### *On the Position of Fractured Limbs.* By Dr. Kinglake.

* Gustavi Lav. Julian Muhrbeck, *Dissertatio Medica de Variolis Spuriis.*

